# Generation of carbamoyl phosphate synthetase 1 reporter cell lines for the assessment of ammonia metabolism

**DOI:** 10.1111/jcmm.13225

**Published:** 2017-05-30

**Authors:** Yi Wang, Le Chang, Jiahui Zhai, Qiao Wu, Donggen Wang, Yunfang Wang

**Affiliations:** ^1^ Stem Cell and Tissue Engineering Lab Beijing Institute of Transfusion Medicine Beijing China; ^2^ Capital Medical University Youan hospital Beijing China

**Keywords:** Carbamoyl phosphate synthetase 1, Sodium phenylbutyrate, Resveratrol, CRISPR/CAS9, Ammonia, Hepatocellular functions

## Abstract

Both primary hepatocytes and stem cells‐derived hepatocyte‐like cells (HLCs) are major sources for bioartificial liver (BAL). Maintenance of hepatocellular functions and induction of functional maturity of HLCs are critical for BAL's support effect. It remains difficult to assess and improve detoxification functions inherent to hepatocytes, including ammonia clearance. Here, we aim to assess ammonia metabolism and identify ammonia detoxification enhancer by developing an imaging strategy. In hepatoma cell line HepG2, and immortalized hepatic cell line LO2, carbamoyl phosphate synthetase 1 (CPS1) gene, the first enzyme of ammonia‐eliminating urea cycle, was labelled with fluorescence protein *via* CRISPR/Cas9 system. With the reporter‐based screening approach, cellular detoxification enhancers were selected among a collection of 182 small molecules. In both CPS1 reporter cell lines, the fluorescence intensity is positively correlated with cellular CPS1 mRNA expression, ammonia elimination and secreted urea, and reflected ammonia detoxification in a dose‐dependent manner. Surprisingly, high‐level CPS1 reporter clones also reserved many other critical hepatocellular functions, for example albumin secretion and cytochrome 450 metabolic functions. Sodium phenylbutyrate and resveratrol were identified to enhance metabolism‐related gene expression and liver‐enriched transcription factors C/EBPα, HNF4α. In conclusion, the CPS1‐reporter system provides an economic and effective platform for assessment of cellular metabolic function and high‐throughput identification of chemical compounds that improve detoxification activities in hepatic lineage cells.

**Highlights**

1. Carbamoyl phosphate synthetase 1 (CPS1) gene was labelled with fluorescence protein *via* CRISPR/Cas9 system in HepG2 and LO2 cells;
2. In both CPS1 reporter cell lines, the fluorescence intensity is positively correlated with both cellular CPS1 mRNA expression and ammonia elimination, secreted urea, reflecting ammonia detoxification in a dose‐dependent manner;
3. Heterogeneity of hepatocellular function is found in the established cell lines HepG2 and LO2;
4. Hepatic function including ammonia elimination is greatly enhanced by small molecules.

## Introduction

Liver failure remains a dramatic and unpredictable disease with a high mortality rate ranging from 60% to 90%. Many studies have demonstrated that liver failure results in the accumulation of a wide range of toxic substances within the blood. Hepatic encephalopathy (HE) is a serious neuropsychiatric complication of both acute and chronic liver failure. It is associated with a dramatic elevation of ammonia, a serious toxin when in excess. The therapy for HE is largely based on the principle of reducing the production and absorption of ammonia in the gut through administration of pharmacological agents such as rifaximin and lactulose 1. Orthotopic liver transplantation (OLT) is the only curative treatment for HE. However, because of the limited availability of donor organs, alternatives to OLT are increasingly needed.

The extracorporeal cell‐based BAL support system has been thus far developed to bridge liver transplantation or to facilitate liver regeneration with the aim of preventing severe complications caused by liver failure and so improve survival 2, 3, 4, 5. It benefits patients through removal of wastes, while having the potential for metabolic detoxification. Freshly isolated human hepatocytes are the preferred cells for BAL devices, but to obtain sufficient human hepatocytes faces the same difficulty of organ shortage and the limited capability for the cells to expand *in vitro*. Immortalized human hepatocellular carcinoma cell lines and human functional HLCs derived from pluripotent stem cells (embryonic stem cells, ESCs or induced pluripotent stem cells, iPSCs) are also candidate cell sources. Loss of hepatocellular functions or inadequate differentiation of stem cell‐derived hepatic lineage cells are the primary reasons for failure of BALs. A challenge is how to assess extracorporeal detoxification for selection of the proper cells to be used in BALs in order to improve extracorporeal detoxification, particularly with respect to ammonia as a way to improve BALs' effects in clinical use.

The urea cycle in the liver plays a predominant role in ammonia disposal, converting ammonia to the relatively harmless urea for excretion 6. The urea cycle consists of five enzymatic reactions sequentially taking place in the mitochondrial matrix and the cytoplasm of periportal hepatocytes. CPS1 locates in the mitochondria and catalyses the first, rate‐limiting reaction 7, 8. It has been well‐established that defects in the function or expression of CPS1 cause hyperammonemia, which can result in brain damage and even death 9, 10. CPS1‐deficient mice die soon after birth with overwhelming hyperammonemia 11. These observations indicate that regulation of CPS1 expression is crucial for ammonia detoxification. Induction of CPS1 expression could be an effective way to improve hepatic ammonia metabolism, to recover damaged hepatocellular functions and to promote immature HLCs to acquire metabolic functions.

Genetic labelling with a reporter gene through genome editing provided a convenient tool to track endogenous gene expression, mark cell stage and optimize differentiation strategy 12, 13, 14. Programmable sequence‐specific endonucleases that facilitate precise editing of endogenous genomic loci have provided a powerful tool for modifying target genome sequences 12, 15, 16, 17, 18. Compared with two other widely used systems zinc‐finger nucleases (ZFNs) and transcription activator‐like effector nuclease (TALEN), the clustered regularly interspaced short palindromic repeat (CRISPR) and CRISPR‐associated 9 (Cas9) system is markedly easier to design, highly specific, efficient for a reporter knockin 19. In this report, we created a CPS1‐tdtomato reporter cell system *via* CRISPR in a hepatic carcinoma cell line, HepG2, and an immortalized hepatic cell line, LO2. With these reporter cell systems, we were able to visualize CPS1 expression and location. We show that cellular fluorescence intensity is positively correlated with CPS1 expression levels, with ammonia metabolism, and also with other critical hepatocellular functions, including albumin secretion and cytochrome P450 (CYP 450) metabolism. Thus, we can use cell imaging to assess hepatocellular function and identify compounds which promote ammonia metabolism with this reporter cell system. The selected compounds, for example sodium phenylbutyrate (NaPB) and resveratrol, were proven to enhance hepatocellular function. This study provides a simple and efficient method to assess cellular metabolic function and a useful platform for searching chemical compounds that improve cellular ammonia detoxification.

## Materials and methods

### Reagents

L‐Ornithine, sodium benzoate, 5‐azacytidine, NaPB, resveratrol, Vitamin K2 and ammonium chloride were purchased from Sigma‐Aldrich (St. Louis, MO, USA). Other collections of small compounds acting as dopamine D3 receptor inhibitor (43 compounds), targeting mammalian targets of rapamycin (mTOR) pathway (58 compounds), or tumour necrosis factor (TNF) pathway (76 compounds) were synthesized and provided by Dr. Wu Zhong's laboratory from the Beijing Institute of Pharmacology & Toxicology. Dulbecco's modified Eagle's medium (DMEM, with or without Phenol Red) was purchased from Gibco (Grand Island, NY, USA). Foetal bovine serum (FBS) was purchased from Nichirei Biosciences (Tokyo, Japan). Hochest 33342, MitoTracker^®^ Green FM^®^ and lipofectamine^®^ 2000 were purchased from Invitrogen (Carlsbad, CA, USA).

### Cell culture, transfection and flow cytometry selection

The HepG2 and LO2 liver cells were purchased from American Type Culture Collection (ATCC) and maintained in DMEM supplemented with 10% foetal bovine serum. Transfection of 0.5 μg pX330 (Cas9‐sgCPS1) and 3 μg donor plasmid were made into 5 × 10^5^ cells with lipofectamine 2000. Single cells were seeded into each well of a 96‐well plate using the BD FACSAria III platform and were subjected to selection conditions with 1 mg/ml G418 for 2 weeks.

### Construction of the sgRNA plasmid and CPS1 donor plasmid

Cas9 target sites were identified using the online CRISPR design tool (crispr.mit.edu) 19. Briefly, 200 bp DNA sequences of the human CPS1 gene flanking the stop codon were used for designing the sgRNAs. The target sequence of sgRNA (sgCPS1) is 5′‐ AGCTGTGCAGAAATCTCGCA ‐3′. To clone a single Cas9‐sgRNA expressing vector, the pX330 (Addgene catalog no. 42230) expression vector expressing Cas9 and sgRNA was linearized with Bbs I digestion, and gel purified. A pair of annealed oligos (20 bp target sequences) were phosphorylated, annealed and ligated to the linearized pX330. Finally, vectors were sequenced to ensure the presence of the right sequence. Construction of CPS1 donor plasmid was based on plasmid pET32‐2A‐tdTomato‐loxP‐CAG‐neo‐loxP (kindly provided by Dr. Hongkui Deng). The 5′ and 3′ homology arms along with the target region were PCR amplified from the genomic DNA extracted from HepG2 cells. The a/b primers bound within 5′homology and the tdtomato were used to ensure proper site‐specific targeting. This region (2.2 kb amplicon) was PCR amplified and sequenced to ensure in‐frame positioning of the reporter/selection fragment. The primers for 5′and 3′homology arm and a,b primers are shown in TableS1.

### Immunofluorescence staining

For immunofluorescent staining, cells were first fixed with 4% paraformaldehyde, then permeabilized and blocked with blocking buffer (PBS containing 0.25% Triton X‐100, 1% BSA, and 10% donkey serum). Cells were incubated with anti‐CPS1 antibodies (Santa Cruz, TX, USA) at 4°C overnight, followed by secondary anti‐goat antibodies conjugated to Alexa Fluor 488 at room temperature for 1 hr in dark. Cells were counterstained with 4′, 6‐diamidino‐2‐phenylindole (DAPI) for visualization of cell nuclei and observed using VECTRA^®^ imaging system (PerkinElmer, Boston, MA, USA). Addition of appropriate normal IgG antibodies and secondary antibodies were provided as negative controls.

### Extraction of genomic DNA

Cellular DNA was extracted by TIANamp Genomic DNA Kit (Tiangen, Beijing, China) according to the manufacturer's protocol for reporter‐insert confirmation.

### Quantitative PCR

RNA was extracted by RNeasy^®^ Mini Kit (Qiagen, Hilden, Germany), and 1 μg RNA was transcribed into cDNA using ReverTra Ace^®^ qPCR RT Master Mix (Toyobo, Kita‐ku Osaka, Japan). QPCR was performed using THUNDERBIRDTM SYBR^®^ qPCR Mix (Toyobo) with primer sets as shown in TableS1. Data were normalized to the housekeeping gene GAPDH.

### Knockdown of CPS1 expression with small interfering RNA

GenOFF™ siRNAs kit targeting CPS1 was purchased from Guangzhou Riobio Co. Ltd. SiRNA (50 nM) was transfected into cells using riboFECT™ CP Transfection kit (Riobio, Guangzhou, China) according to the manufacturer's protocol.

### Hepatic functional assay including albumin secretion, ammonia determination, urea secretion and cellular CYP3A4 activity

Cells were cultured in the medium without phenol red. Culture supernatants were collected 24 hrs after medium changes. Albumin, ammonia and urea concentration in the supernatants were analysed using the human albumin ELISA kit (Bethyl Laboratories, Montgomery, TX, USA ), ammonia assay kit (Sigma‐Aldrich) and the QuantiChrom Urea Assay Kit (BioAssay Systems, Hayward, CA, USA) according to each manufacturer's instructions. Cellular CYP3A4 activity was quantitated with P450‐Glo™ CYP3A4 Screening System with Luciferin‐IPA (Promega, Madison, WI, USA) according to the manufacturer's instructions.

### Cell viability assay

The viability of cells was evaluated using a Cell Counting Kit‐8 (Applygen, Beijing, China). The reduction of WST‐8 was measured photometrically using a SpectraMax M5 (Molecular Devices, Sunnyvale, CA, USA) at 450 nm.

### Cells treatment with small compounds and high‐throughput imaging analysis

Cells were grown in 96‐well plate (Black with clear flat Bottom, Costar) in DMEM containing 10% foetal bovine serum, and were cultured in an atmosphere containing 5% CO_2_ at 37°C overnight. Then the culture media were changed to DMEM (no phenol red) containing various small molecules. Before imaging, cells were incubated with Mitotracker dye and Hochest 33342 for 10 min. at 37°C. To investigate the correlation between the fluorescence intensity of cell with other ammonia metabolism parameters, the fluorescence data and images of cells were acquired by IN Cell Analyzer 2000 (GE Healthcare, Uppsala, Sweden) and expressed as intensity/cell. For high‐throughput screening, the fluorescence data expressed as median intensity/well and images of cells were collected by Ensight™ multiple plate reader (PerkinElmer, Boston, MA, USA).

### Statistical analysis

The results from two experiments are expressed as the mean ± S.E.M. The data are analysed using a one‐way anova followed by Student‐Newman‐Keuls Method. *P*‐values less than 5% levels were considered statistically significant.

## Results

### Establishment of fluorescence reporter system in HepG2 and LO2 cell lines

**Figure 1:**
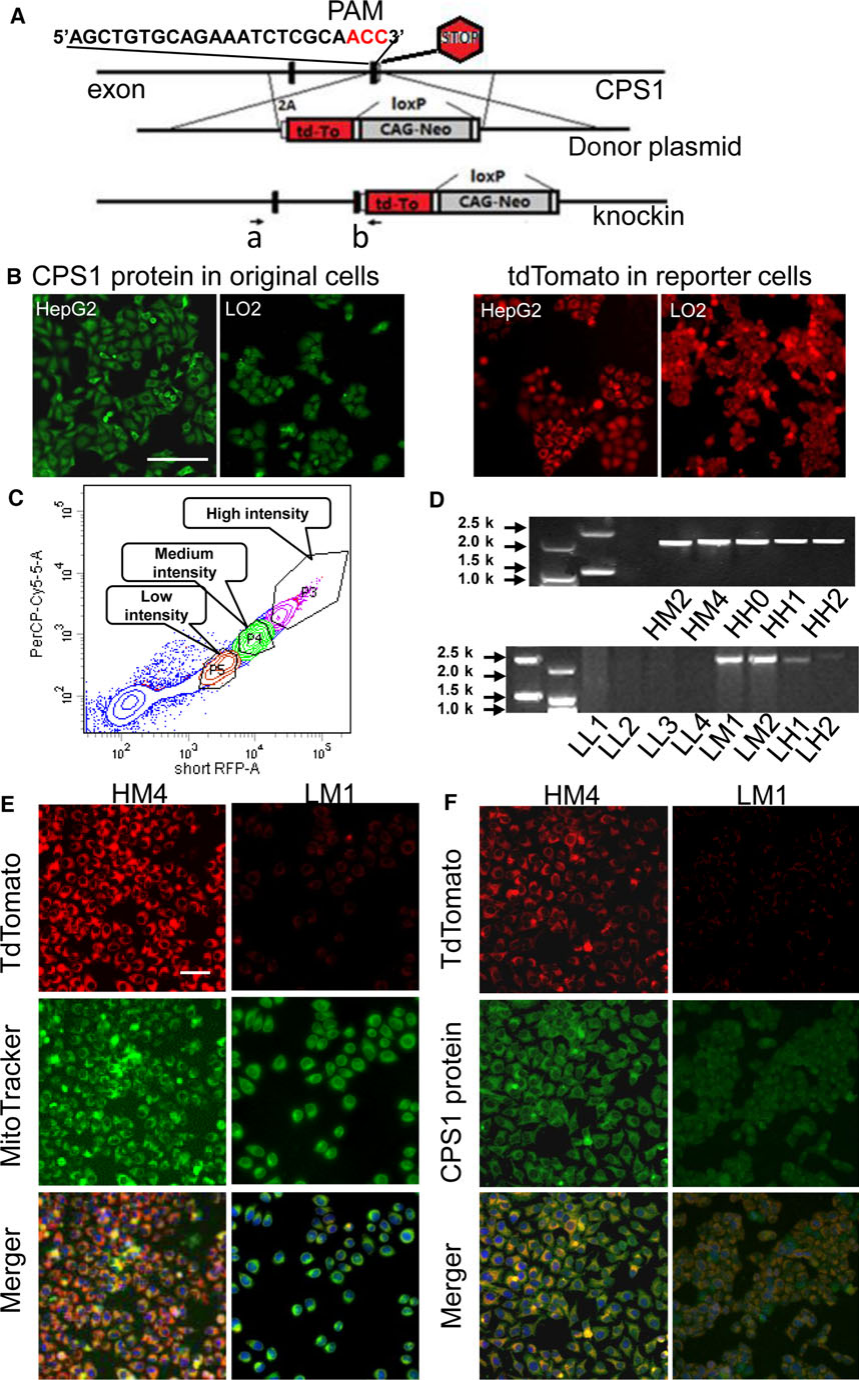
Targeting of tdTomato fluorescence gene into CPS1 loci in liver cell line. (**A**) Diagram of the knockin strategy. (**B**) CPS1 expression in original liver cell lines and general fluorescent imaging of reporter cells HepG2‐CPS1‐tdTm (HCT) and LO2‐CPS1‐tdTm (LCT) derived from HepG2 and LO2 cells. Scale bar: 100 μm. (**C**) Fluorescence histograms of reporter cells HCT and LCT using flow cytometry analysis. P3→P5: subpopulation of reporter cells with decreasing intensity. (**D**) Identification of the reporter clones with the primer set a+b. To facilitate the description of these clones, each clone was named ‘parent cell name‐intensity‐clone number'. For instance, ‘HM1' stands for HepG2 derived clone 1 with medium intensity. (**E**) Colocalization of tdTomato fluorescence signal and mitochondria indicated with MitoTracker^®^ Green FM^®^ in reporter cells. (**F**) Colocalization of tdTomato fluorescence signal and CPS1 protein detected with immunostaining in reporter cells. Scale bar: 50 μm.

To create the reporter system, we transfected HepG2 or LO2 cells *via* lipofectamine with two plasmids: a CRISPR/CAS9 plasmid containing CAS9 and sgCPS1 targeting the 61 bases in front of the CPS1 stop codon and homologous recombination donor plasmid. The donor plasmid contained two tdtomato‐flanking homology arms to CPS1, a 2 kilobase (kb) left arm and a 2.3 kb right arm. CRISPR‐mediated insertion of the 2A‐tdtomato‐loxP‐CAG‐Neo‐loxP sequence into the endogenous CPS1 locus and replaced the CPS1 stop code (Fig. 1A). The distribution of fluorescence signal demonstrated a similar pattern to that of CPS1 expression (Fig. 1B). The cells were divided into three populations dictated by the intensity of fluorescence using flow cytometry and seeded into a 96‐well plate as a single cell/well (Fig. 1C). Transfected cells were selected with 1 mg/ml G418 for 2 weeks. In the tdtomato positive clones, red fluorescence protein was found to occur in different locations: in the cytoplasm or throughout the whole cell (Fig. 1B). Those clones with a red signal in the cytoplasm were picked. The CPS1‐tdTomato reporter clones were selected with PCR and confirmed with costaining with MitoTracker^®^ and CPS1 antibody (Fig. 1D, E and F). The PCR products were purified (Fig. 1D), and sequenced to confirm correct insertion of 2A‐tdTomato‐loxP ‐CAG‐Neo‐loxP. Taken together, the experiments suggested successful establishment of a fluorescence reporter system in liver cell lines.

### Characterization and quantization of CPS1‐tdTomato reporter liver cell clones

**Figure 2:**
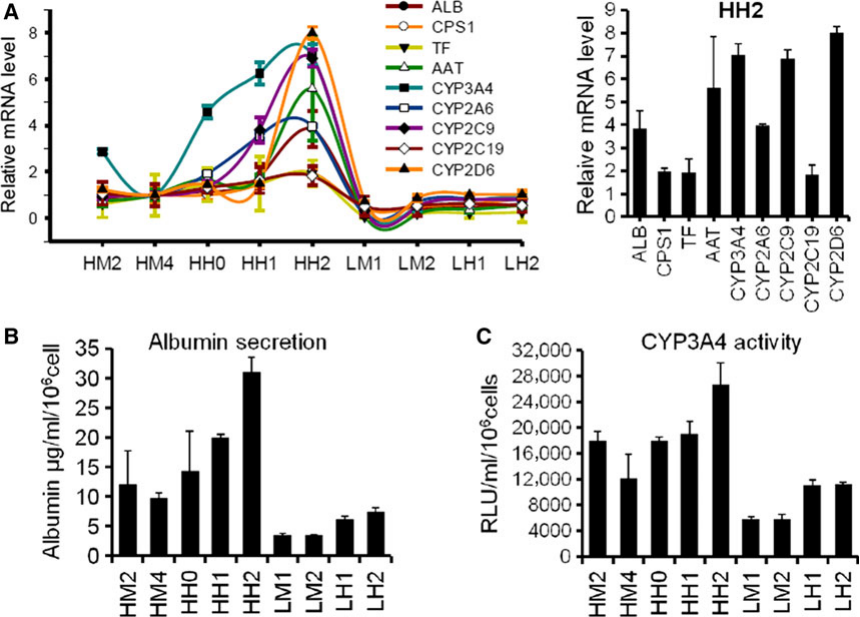
Characterization and quantization of CPS1‐tdTomato reporter liver cell clones. (**A**) qRT‐PCR analysis of different CPS1‐tdTomato reporter clones derived from HepG2 and LO2. Relative mRNA levels of each gene in HH2 were shown in the right panel as a bar histogram. (**B**) Quantitative analysis of human albumin secreted by each clone over 24 hrs. (**C**) CYP3A4 activity from each clone detected by P450‐Glo™ assays.

The labelled cells expressed different levels of fluorescence signal. We wondered about the hepatic functions of the cells with different signal intensities. The mRNA levels of different representative genes of classical hepatic functions were assessed using qRT‐PCR. The reporter clones with strong signal intensity expressed high‐level CPS1 and also high‐levels of other hepatic functions (Fig. 2). The clones from HepG2 showed rather higher levels of hepatic gene expression compared with those from LO2 (Fig. 2A). Corresponding to these results, the secreted albumin and cellular CYP3A4 activity were higher in those cells with stronger intensities (Fig. 2B and C), which indicated that those cells with high‐level CPS1 expression also possessed higher levels of other critical hepatic functions.

### Linear correlation between fluorescence intensity, cellular CPS1 mRNA and ammonia detoxification

**Figure 3:**
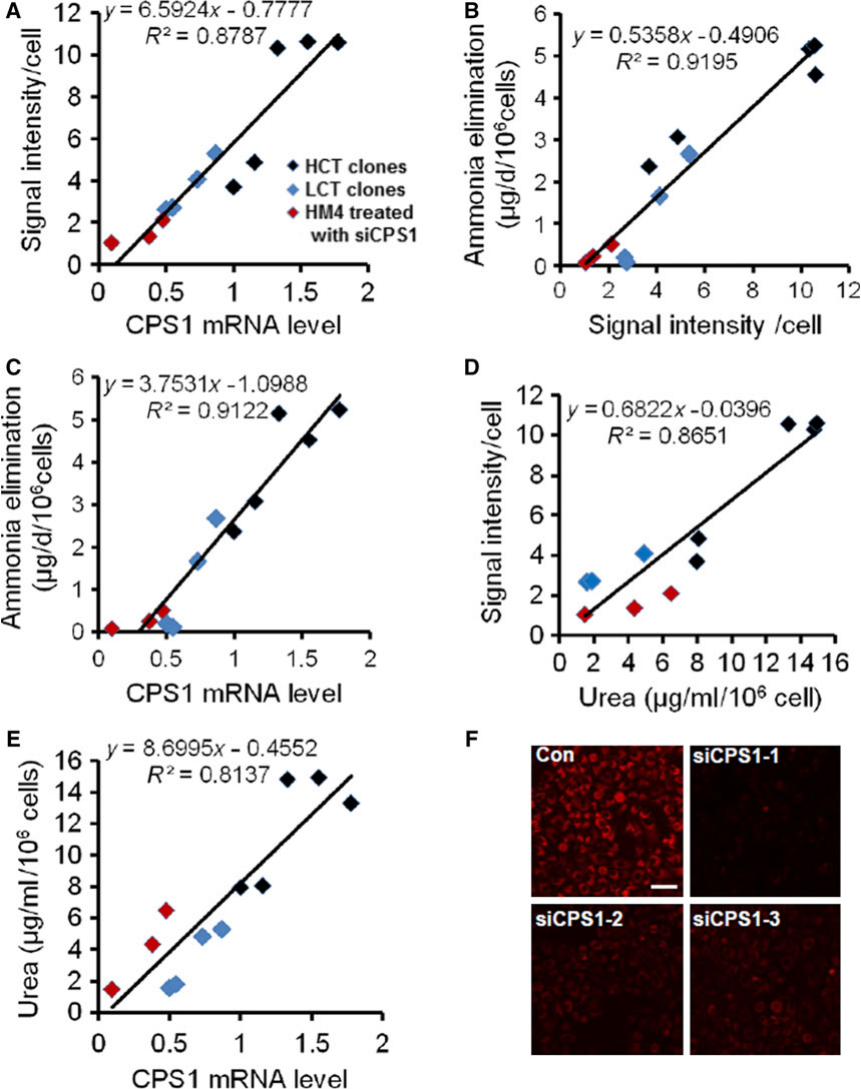
Correlation among fluorescent intensity, CPS1 expression, ammonia elimination and synthesize urea in liver cells. (**A‐E**) Cells of different HCT and LCT clones were seeded into 96‐well clear bottom black plate. Cell images were captured and fluorescent intensity of each cell was analysed with GE 2000 automated high‐content cell imaging system. The cells from each clone were collected for analysis of CPS1 mRNA level. The supernatant were collected for ammonia elimination and urea concentration determination. Each point accounts for cells from one clone. The continuous line represents the least square fit linear regression, and the related goodness of fit coefficient (*R* ^2^) is indicated on each graph. (**F**) Representative image of HepG2 reporter cells M4 transfected with different siRNA targeting CPS1.Scale bar: 50 μm.

To investigate whether the reporter system can be used for screening of compounds to improve cellular ammonia metabolism, we needed to confirm the correlation between fluorescence intensity and ammonia detoxification. We quantified the fluorescence intensity of each cell from each selected clone with an IN Cell Analyzer 2000 high‐content imaging system. We also collected the supernatant for ammonia elimination and urea concentration measurement, and cell lysate for CPS1 mRNA quantization. We combined and analysed the data from HepG2 and LO2 clones. The results showed that a linear correlation exist not only between fluorescence intensity and cellular CPS1 mRNA (Fig. 3A, correlation coefficient *R* ^2^ = 0.8787), ammonia elimination (Fig. 3B, *R* ^2^ = 0.9195) and urea production (Fig. 3D, *R* ^2^ = 0.8651), but also between cellular CPS1 mRNA and ammonia elimination (Fig. 3C, *R* ^2^ = 0.9122) and urea production (Fig. 3E, *R* ^2^ = 0.8137). The LO2 clones generally had lower CPS1 expression than the HepG2 clones and showed less ammonia elimination and synthesized urea. We also validated that this correlation existed in one clone. We use siRNAs targeting CPS1 in HepG‐CPS1‐tdTm (HCT) cells (clone M4) and observed their effect on cellular fluorescence intensity. We set the mRNA of CPS1 in HCT clone M4 as 1. Quantitative PCR result showed the suppression efficiency of siCPS1‐1 was best and the CPS1 expression was inhibited up to 90% (Fig. 3F). The suppression effect of siCPS1 on fluorescence intensity also confirmed the correct insertion of the reporter tag. The correlation of the fluorescence intensity, secreted urea and CPS1 mRNA levels were found to exist in siCPS1‐treated cells. These data indicate that the cellular fluorescence signal can directly reflect cellular ammonia metabolism.

### Screening of small molecules that could act as enhancers of cellular ammonia detoxification

**Figure 4:**
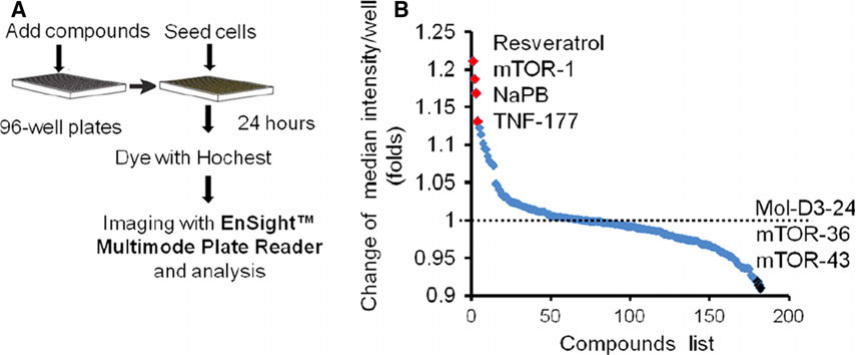
Small compounds screening targeting CPS1 regulation with reporter cells. (**A**) A schematic of the chemical screening platform. (**B**) A plot indicating the results from assays on 180 small molecules screened for their activity of CPS1 regulation. Highlighted dots filled with red were labelled as representative compounds that showed increase of CPS1 expression. Highlighted dots filled with black were labelled as representative compounds suppressing CPS1 expression. The dotted line showed the signal intensity of control.

Based on the above results, the cellular ammonia metabolism was able to be visualized directly through cell imaging in the CPS1 reporter system. We used the high‐throughput imaging system to observe the effects of small molecules on cellular fluorescence intensity and to identify potential enhancers of cellular ammonia detoxification. HCT cells (clone M4) were seeded in 96‐well black plates with clear flat bottoms. After an overnight recovery, the DMEM medium was changed to be DMEM (with no phenol red) supplemented with individual compounds (1 μM). After 24 hrs, cells were stained with Hochest 33342, and imaged using an Ensight™ multiple plate reader with fourfold imaging system for the primary analysis of cytoplasmic fluorescence intensity in each well (Fig. 4A).

**Figure 5:**
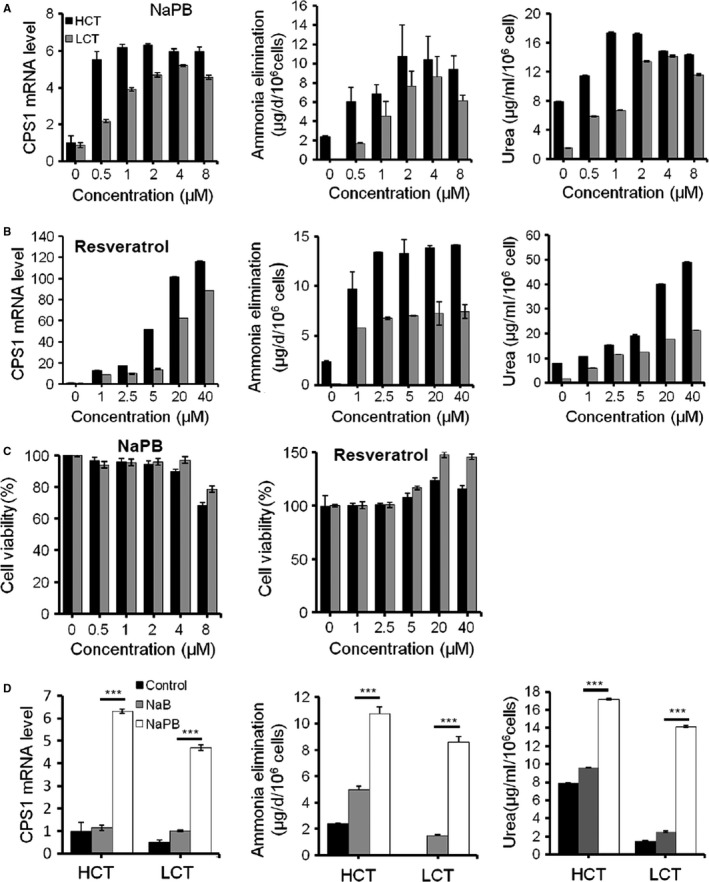
The dosage effects of NaPB, resveratrol and NaB on cellular ammonia metabolism. (**A‐C**) The dosage effects of NaPB and resveratrol on CPS1 expression, ammonia elimination, urea concentration, and cell viability as assessed by cell counting, of HCT and LCT cells 24 hrs after the addition of different doses of NaPB and resveratrol.(**D**) Ammonia metabolism in HCT and LCT cells treated with NaB and NaPB for 24 hrs. The concentration of compounds was 2 μM for HCT cells, 4 μM for LCT cells. ***P < 0.001.

From a collection of 182 small molecules consisting of compounds approved or under investigation for hyperammonemia treatment (*e.g*. Sodium benzoate (NaB), NaPB, L‐ornithine, 5‐azacytidine and resveratrol) 6, 20, 21, 22, 23, 24, dopamine D3 receptor inhibitor, mTOR and TNF pathway activator/inhibitor, 23 small molecules increased the signal intensity, while 21 repressed the fluorescence intensity significantly. The representative positive hits are shown in Figure 4B. Consistent with the decreasing signal, the cells became smaller over 20 days after treatment with certain chemical compounds (data not shown). We confirmed two ammonia clearance enhancer, NaPB and resveratrol, with other functional parameters. NaPB improved the secreted urea concentration in both HepG2 and LO2 cells at 24 hrs. NaPB showed similar effects above 2 μM but with serious toxicity at 8 μM in HepG2 cells. NaPB achieved maximal effects at 4 μM in LO2 cells (Fig. 5A). Notably, at their optimized concentrations, the compounds exhibited no or very mild toxicity as assayed by both cell counts and CCK‐8 cell proliferation assay (Fig. 5C). Compared with another ammonia scavenger NaB, NaPB exhibited more enhancement on CPS1 expression and ammonia clearance (Fig. 5D) Resveratrol‐enhanced cellular ammonia detoxification dose a dose‐dependent manner at a wider concentration range (1–40 μM), and no cellular toxicity was found (Fig. 5B and C).

### Upregulation of hepatic functional genes and transcription factor C/EBPα, HNF4α, FOXO3a by NaPB and resveratrol

**Figure 6:**
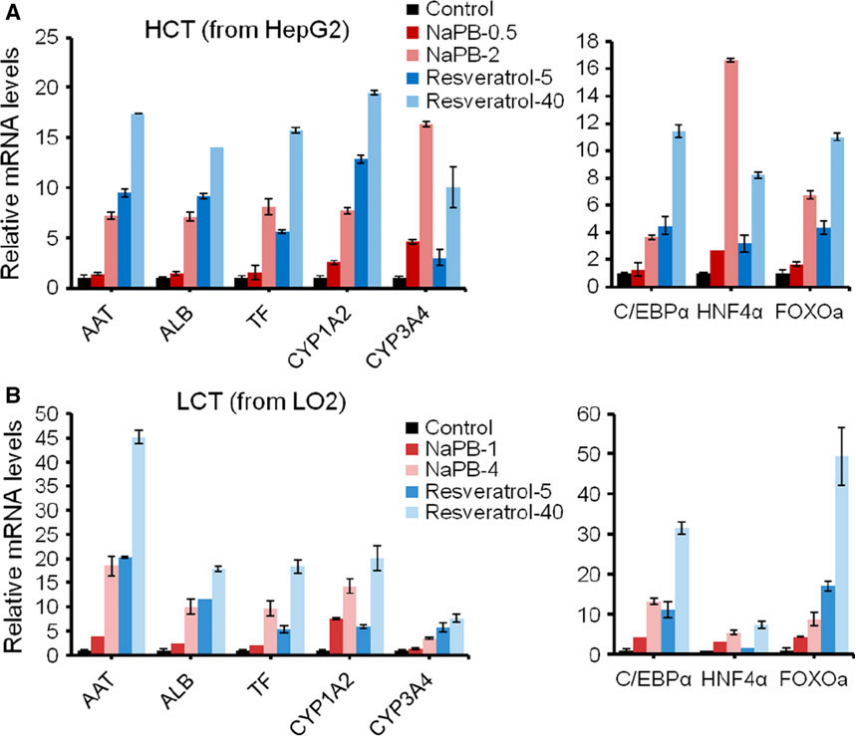
Improved functional gene expression and transcription factors by NaPB and resveratrol in liver cell lines. (**A‐B**) qRT‐PCR analysis of representative functional genes and transcription factors in HCT and LCT cells treated with different doses of NaPB and resveratrol.

To investigate the effect of NaPB and resveratrol on other critical hepatic function, we detected several representative genes including AAT, ALB, TF, and CYP450 family genes. Both NaPB and resveratrol could increase functional gene expression in dose‐dependent manner (Fig. 6A and B). In HepG2 cells, the elevated fold for each gene triggered by resveratrol was larger than that triggered by NaPB, except CYP3A4. NaPB (2 μM) increased CYP3A4 expression by about 16‐fold, while 12‐fold in the cells treated with 40 μM resveratrol. To find the mechanism involved CPS1 expression regulation, we detected two liver‐enriched key transcription factors, CCAAT enhancer‐binding protein‐alpha (C/EBPα) and hepatocyte nuclear factor 4‐alpha (HNF4α) in HepG2 and LO2 cells treated by NaPB and resveratrol. HNF4α is the core hepatic transcript factor and plays an important role in liver development 25. C/EBPα is considered as a hepatic maturation factor 26. Analysis of the CPS1 promoter revealed that C/EBPα was one of the cis‐regulatory elements required for the maximum promoter activity. C/EBPα‐deficient mice lack CPS1 expression and exhibit hyperammonemia, indicating that C/EBPα is essential for CPS1 expression 27. C/EBPα was activated by both small molecules, much more by resveratrol, consisted with enhanced ammonia detoxification. HNF4α was also upregulated by both compounds. Compared with C/EBPα, NaPB increased HNF4α more, indicating NaPB played a more important role in hepatic specification than in hepatic maturation. We also detected another transcription factor Foxo3a, a downstream molecule of resveratrol‐sirt. Resveratrol improved Foxo3a expression, which could explain that resveratrol had no cytotoxicity but promote cell proliferation by 50% at 40 μM. NaPB also improved Foxo3a expression, but it showed slight cytotoxicity at its optimal concentration. Taken together, NaPB and resveratrol enhanced hepatic functional gene expression through C/EBPα and HNF4α.

## Discussion

The liver is one of the main detoxifying organs of the body. The maintenance of hepatocellular functions is critical for clearance of metabolic toxins including ammonia, which is essential for a life‐saving BAL device. Establishment of a simple platform for assessment of cellular metabolic function and optimization of candidate cells functionality is useful to select proper cells for a BAL. The present study established a CPS1 reporter system *via* CRISPR and assessed hepatic functions of different liver cells through cell imaging.

Using the CRISPR/Cas system, we have demonstrated a highly efficient approach for generation of the knock‐in reporter in human liver cell lines. Based on this precise genomic modification, the reporter tdtomato gene was inserted at the stop code position of CPS1 in the chromosome. The expression of CPS1 and tdtomoto gene is under the CPS1 promoter. Thus, the fluorescence intensity of tdtomoto can represent CPS1 expression and reflect the hepatic function inherent to each cell among the same cell lines. In these reporter cells, CPS1 expression became *visible* and traceable. As is the case for many mitochondrial proteins, CPS1 is synthesized in the cytoplasm as a precursor that is ~5 kD larger than the mature, active protein. The precursor contains an N‐terminal signal peptide of 38 residues, which is required for importing the protein into the mitochondria 28.We chose those cells with active CPS1, that is with fluorescence signal only in the cytoplasm, for the tests. How to transform the inactive form into an active one will also be a good way to improve the cellular urea cycle and will be the focus of future investigations.

Further functional analysis of different CPS1‐tdtomato clones indicated that cell heterogeneity in metabolic functions existed in each cell line. Clones expressing high‐levels of CPS1 also secreted more albumin and had high CYP3A4 enzymatic activity, which made these cells optimal for BALs. The variability in tissue‐specific functions of cultured hepatocytes makes them uncertain as cells to be used for BALs. CRISPR system worked as earlier as 24 hrs after transfection of sgRNA and donor plasmids 29.Using the reporter clones can facilitate identifying those primary hepatocytes that will be useful 30, 31. Thus, monitoring CPS1 expression is a good way to identify cells that are optimal for use in BALs.

For successful application of the reporter system to the selection of ammonia‐detoxification compounds, correlation between signal intensity and ammonia metabolism should be established. The correlation was found among different clones form both HepG2 and LO2 cells, which indicated the reliability of the system. Combined with the high‐throughput imaging and data collection, this reporter can be used for high‐throughput screening. NaPB, tested in the system, is a clinical drug used as an ammonia sink 20, 21. It is the prodrug of phenylacetate. It also acted as a chemical chaperone and a histone deacetylase 21. NaPB has been reported to activate many genes involved in cell differentiation and apoptosis. Compared with LO2 cells, HepG2 cells were sensitive to NaPB. This result might be connected with NaPB's antitumour effect through cellular apoptosis induction. CPS1 activation by NaPB reported here was a novel mechanism for ammonia clearance. Several research reports have shown that NaPB partially restored bile salt export pump (BSEP) expression at the canalicular membrane and significantly improved liver functions in patients with progressive familial intrahepatic cholestasis type 2 32, 33. We also found that NaPB improved hepatic differentiation related factor HNF4α and liver maturation factor C/EBPα. Several *in vivo* and *in vitro* studies provided evidence for the potential role of resveratrol as a hepatoprotective agent mainly through activation of LKB1‐AMPK and sirt1 pathway 24. Resveratrol was also reported to activate sirt5 which deacelyated CPS1 and increased CPS1 activity, indicted that resveratrol could enhance CPS1 activity through post‐translation modification 34. Our data showed resveratrol might also be involved in transcription regulation of CPS1 and other liver function genes. It had the potential function as a hepatic function enhancer through its positive effect on C/EBPα and HNF4α expression. Many proteins involved liver function including CPS1, CYP450 exist and played their roles in mitochondria. Resveratrol was an important inducer for mitochondria biogenesis 35, which could be another reason for resveratrol as hepatic function enhancer. The net sum of these results indicates that NaPB and resveratrol could be promising candidates driving differentiation and function maturation of hepatocytes. The consistence among the diminished signal, repression of functional gene CPS1 expression and cell morphology change, activation of stemness‐related genes over time also made such a platform suitable for the selection of stem‐like cells proliferation enhancer.

In conclusion, we established a reporter system to visualize the cellular urea cycle with fluorescence imaging. Our study provides a simple and efficient approach to assess cellular metabolic functions. We also provide a platform to search chemical compounds that enhance ammonia metabolism. Because CPS1 is regarded as an important marker of hepatocellular functions, the compounds selected with this platform may also be used to maintain primary hepatocytes or promote stem cell‐derived HLCs metabolic functional maturation and to improve BAL effects in clinical application.

## Conflict of interest

The authors confirm that there are no conflicts of interest.

## Supporting information


**Table S1** PCR primer lists.
