# Supplementary material for: Generation of carbamoyl phosphate synthetase 1 reporter cell lines for the assessment of ammonia metabolism
Source: J Cell Mol Med. 2017 May 30;21(12):3214–23. doi: 10.1111/jcmm.13225 (PMC5706564; doi:10.1111/jcmm.13225)
Supplement: Supplementary file 1 — Table S1 PCR primer lists. [file JCMM-21-3214-s001.doc]

**SUPPLEMENTAL INFORMATION**

**Generation of Carbamoyl Phosphate Synthetase 1 Reporter Cell Lines for the Assessment of Ammonia Metabolism**

Yi Wang^1,3^, Le Chang^1,3^, Jiahui Zhai^1^, Qiao Wu^2^, Donggen Wang^1^ & Yunfang Wang^1^

^1^. Stem Cell and Tissue Engineering Lab, Beijing Institute of Transfusion Medicine, Beijing, China 100850

^2^. Capital Medical University Youan hospital, Beijing,China 100069

^3^. Co-first authors

Corresponding author:

Yunfang Wang, Ph.D, MD

Director, Stem Cell and Tissue Engineering Lab, Beijing Institute of Transfusion Medicine,

27(9) Taiping Road, Beijing, China 100850

Phone: (8610)66930920

Email:wangyf1972@gmail.com

Co-corresponding author

Donggen Wang, MD

Director, Beijing Institute of Transfusion Medicine, Beijing, China 100850

Phone: (8610)66931980

Email:13901183367@139.com

Supplementary table

**Table1. PCR primer lists**

|  | Gene | Primers (5’-3’) |
| --- | --- | --- |
| Real-time PCR | AAT | F: ATGCTGCCCAGAAGACAGATA  R: TTGTTGAAGGTTGGGTGATCC |
|  | ALB | F:GCAGTGTCCATTTGAAGATCATGTA  R:TGCAACTGTGCATAATTTGTCTCC |
|  | CPS1 | F:AAGCCACATCAGACTGGCTCA  R:TCACTAGGTCAATGCTGCCATCTC |
|  | TF | F:TGGCCTTTGTGAAACACCAGAC  R:TACCATCAAGGCACAGCAACTCAT |
|  | CYP1A2 | F: ATGGCATTGTCCCAGTCTGTT  R: TGGCTCTGGTGGACTTTTCAG |
|  | CYP2C9 | F:GGACAGAGACGACAAGCACA  R:CATCTGTGTAGGGCATGTGG |
|  | CYP2C19 | F:GAAGAGGAGCATTGAGGACCG  R:GCCCAGGATGAAAGTGGGA |
|  | CYP2D6 | F:GAAGGATGAGGCCGTCTGGG  R:GGAAAGCAAAGACACCATGG |
|  | CYP3A4 | F: AAGTCGCCTCGAAGATACACA  R: AAGGAGAGAACACTGCTCGTG |
|  | C/EBPα | F:GTGGACAAGAACAGCAACGA  R: GGTCATTGTCACTGGTCAGC |
|  | HNF4α | \| F:ACGGACAGATGTGTGAGTGG \| \| --- \| \| R:CAGGAGCTTATAGGGCTCAGA \| |
|  | FOXO3a | F: CGCACGTCTTCAGGTCCTC  R: AGCACCAAAGAAGAGAGAAGGA |
|  | GAPDH | F:CATGAGAAGTATGACAACAGCCT  R:AGTCCTTCCACGATACCAAAGT |
| sgRNA | sgCPS1 | F:CACCGAGCTGTGCAGAAATCTCGCA  R:AAACTGCGAGATTTCTGCACAGCTC |
| Donor plasmid | CPS1 left arm | F:GAAGATCTTTGTGTGAATCTTCAGGAATA  R:CGGATATCTGCTGCTTTTCCAGCACTGT |
|  | CPS1 right arm | F:ACGCGTCGACAGATGCAGACACCCCAGCC  R:CCCAAGCTTGAAGTAATGAAAGTCTTGAC |
|  | Clone identification | F:TTGGGGCATTGTAAAACAAT  R:CCGGTGCCATGCCCCAGGAA |
